# Supplementary material for: COZOID: contact zone identifier for visual analysis of protein-protein interactions
Source: BMC Bioinformatics. 2018 Apr 6;19:125. doi: 10.1186/s12859-018-2113-6 (PMC5889581; doi:10.1186/s12859-018-2113-6)
Supplement: Supplementary file 2 — Software build. Executable binary file of the software tool. (ZIP 118,784 kb) [file 12859_2018_2113_MOESM2_ESM.zip › cozoid/java/docs/beansbinding-1.2.1-doc.zip!/org/jdesktop/beansbinding/Converter.html]

Converter (Beans Binding)


---


|  |  |  |
| --- | --- | --- |
| **Overview** \| **Package** \| **Class** \| **Use** \| **Tree** \| **Deprecated** \| **Index** \| **Help** | |  |
| **PREV CLASS**   **NEXT CLASS** | **FRAMES**    **NO FRAMES**     **All Classes** |  |
| SUMMARY: NESTED \| FIELD \| CONSTR \| METHOD | DETAIL: FIELD \| CONSTR \| METHOD |  |


---


## org.jdesktop.beansbinding Class Converter<S,T>

```
java.lang.Object
  org.jdesktop.beansbinding.Converter<S,T>
```

**Type Parameters:**: `S` - the `Converter's` source type: `T` - the `Converter's` target type

---

``` public abstract class Converter<S,T> extends java.lang.Object ```

`Converter` is responsible for converting a value from one type
to another.

The conversion methods can throw `RuntimeExceptions` in response
to a problem in conversion. For example, a `String` to `Integer`
converter might throw a `NumberFormatException` if the `String`
can't be parsed properly into an `Integer`.

---

**Constructor Summary**

`Converter()`


| **Method Summary** | |
| --- | --- |
| `abstract  T` | `convertForward(S value)`             Converts a value from the source type to the target type. |
| `abstract  S` | `convertReverse(T value)`             Converts a value from the target type to the source type. |

**Methods inherited from class java.lang.Object**

`clone, equals, finalize, getClass, hashCode, notify, notifyAll, toString, wait, wait, wait`

**Constructor Detail**

### Converter

```
public Converter()
```


**Method Detail**

### convertForward

```
public abstract T convertForward(S value)
```

:   Converts a value from the source type to the target type.
    Can throw a `RuntimeException` to indicate a problem
    with the conversion.

    :   **Parameters:**: `value` - the source value to convert **Returns:**: the value, converted to the target type

---


### convertReverse

```
public abstract S convertReverse(T value)
```

:   Converts a value from the target type to the source type.
    Can throw a `RuntimeException` to indicate a problem
    with the conversion.

    :   **Parameters:**: `value` - the target value to convert **Returns:**: the value, converted to the source type


---


|  |  |  |
| --- | --- | --- |
| **Overview** \| **Package** \| **Class** \| **Use** \| **Tree** \| **Deprecated** \| **Index** \| **Help** | |  |
| **PREV CLASS**   **NEXT CLASS** | **FRAMES**    **NO FRAMES**     **All Classes** |  |
| SUMMARY: NESTED \| FIELD \| CONSTR \| METHOD | DETAIL: FIELD \| CONSTR \| METHOD |  |


---
